# Acute Normobaric Hypoxia at 2000 and 3000 m Significantly Lowers the Maximal Lactate Steady State (MLSS) in Trained Cyclists: Training Implications

**DOI:** 10.5114/jhk/217391

**Published:** 2026-04-02

**Authors:** Marta Bazańska-Janas, Natalia Grzebisz-Zatońska, Kamila Płoszczyca, Marek Janas, Adam Niemaszyk, Miłosz Czuba

**Affiliations:** 1 VO2MAX Sp. z o.o., Wólka Radzymińska, Poland.; 2 Faculty of Rehabilitation, Józef Piłsudski University of Physical Education in Warsaw, Warsaw, Poland.; 3 Department of Applied and Clinical Physiology, Collegium Medicum University of Zielona Gora, Zielona Góra, Poland.

**Keywords:** altitude, athletes, hypoxia, performance, heart rate, workload

## Abstract

The reduced availability of oxygen under hypoxic conditions makes it necessary to adjust training intensity by reducing the workload and modifying exercise zones. The aim of this study was to evaluate the effects of normobaric hypoxia of varying severity (H2000: FiO_2_ = 16.5%, ~2000 m; H3000: FiO_2_ = 14.5%, ~3000 m) on physiological variables associated with the maximal lactate steady state (MLSS) in trained cyclists, including the work rate (WRMLSS), oxygen uptake (VO_2_MLSS), minute ventilation (V̇EMLSS), and the heart rate (HRMLSS). Sixteen trained male cyclists (age: 30 ± 5 years; body height: 180.5 ± 8.0 cm; body mass: 75.2 ± 9.0 kg; body fat content: 10.2 ± 2.0%; VO_2__max_: 57.0 ± 6.0 ml/kg/min) performed incremental and constant-load exercise tests under normoxic (N) and hypoxic (H2000 and H3000) conditions to determine the MLSS. Exposure to H2000 and H3000 significantly reduced the WRMLSS by 9.3% and 18.5%, respectively, as well as VO_2_MLSS by 7.2% and 17% compared with normoxia (p < 0.05), while the HRMLSS remained unchanged. SpO_2_ showed a statistically significant (p < 0.05) drop between N and H2000 (−10.61%), as well as N and H3000 (−16.43%). Blood lactate concentration at the 30^th^ min of MLSS exercise was significantly (p < 0.05) higher under both H2000 (+36%) and H3000 (+34%) conditions compared to N. These findings indicate that acute normobaric hypoxia equivalent to 2000 and 3000 m significantly impairs the ability to sustain exercise at MLSS intensity. It is recommended that MLSS power be reduced by ~10% at 2000 m and by ~20% at 3000 m, while HR values remain unchanged under both conditions.

## Introduction

In contemporary sports training, across a wide range of disciplines, including cycling, hypoxia (either normobaric or hypobaric) has now become a standard component of athletes’ conditioning programs. These methods are designed to enhance performance at the sea level or to facilitate acclimatization before competitions held at high altitudes or during the altitude ascent (Płoszczyca et al., 2018). Exposure to hypoxic conditions, whether at rest or during exercise, triggers a range of adaptive physiological responses aimed at maintaining homeostasis, which in turn can boost the effectiveness of conventional training methods (Niemaszyk et al., 2025).

Adaptation of the human body to hypoxia is primarily regulated by the hypoxia-inducible transcription factor (HIF), which activates genes responsible for favorable hematological and non-hematological changes necessary for coping with oxygen deficiency (Płoszczyca et al., 2018). In response to HIF activation, several adaptations occur, including accelerated erythropoiesis, expansion of the capillary network, increased mitochondrial density, improved buffering capacity of muscle tissue, and elevated activity of glycolytic enzymes—all of which significantly enhance physical performance (Wiśniewska et al., 2020). Additional effects include reduced energy cost of exercise and improved exercise capacity under both normoxic and hypoxic conditions (Czuba et al., 2014).

It has been shown that, except for static exercises and short-term high-intensity exercise (Perrey 2009), hypoxia contributes to reduced exercise capacity (Chapman et al., 2014). The direct cause for the reduction of exercise capacity in hypoxia is the reduction of maximal oxygen uptake (VO_2max_). Diminished VO_2max_ in hypoxia is accompanied by a lowered O_2_ partial pressure in arterial blood (PaO_2_), which reduces O_2_ delivery to tissues and negatively affects muscle metabolism and contraction (Chapman et al., 2014; Wehrlin and Hallen, 2006). This effect is associated with the exhaustion of energy stores in skeletal muscles and the build-up of metabolic by-products (Bigland-Ritchie et al., 1986).

Currently, cycling training primarily employs moderate hypoxic stimuli corresponding to altitudes between 2000 and 3000 m a.s.l. (Czuba et al., 2011, 2018; Hodun et al., 2024; Michalczyk et al., 2019; Płoszczyca et al., 2021). Higher levels of hypoxia are associated with a significantly greater impairment of exercise capacity, which may result in reduced effectiveness of such training (Czuba et al., 2014, 2018). Dempsey and Wagner (1999) observed that each 1% decrease in hemoglobin saturation (SpO_2_) below 95% contributed to a 1–2% decrease in VO_2max_. In other words, an increase in the altitude by each 1000 m above the sea level (a.s.l.) leads to a reduction in VO_2max_ by ~8% (Wehrlin and Hallen, 2006). As a result, at moderate altitudes (2000–3000 m a.s.l.), VO_2__max_ is 10–20% lower; however, significant interindividual differences are observed within this range (Lawler et al., 1988). It has been repeatedly demonstrated that athletes with high cardiovascular fitness are more susceptible to a decrease in VO_2__max_ with the increasing altitude compared to untrained individuals (Mollard et al., 2007). Moreover, the literature also reports that in individuals sensitive to hypoxia, including athletes, VO_2__max_ may decrease even at altitudes below 1000 m a.s.l. (Wehrlin and Hallen, 2006).

As outlined above, the impact of hypoxia on the reduction of VO_2__max_ has been relatively well documented in the literature. However, data regarding the effects of hypoxia on the anaerobic threshold and exercise intensity zones remain limited. Accurately defining training intensity zones is crucial for structuring endurance training and monitoring adaptations, both under normoxic conditions and in increasingly popular hypoxic training (Faiss et al., 2013; Matomäki, 2025).

Low-intensity training forms the foundation of aerobic endurance development, supporting recovery and promoting key adaptations such as increased mitochondrial biogenesis, capillarization, oxidative enzyme activity, and the ability to use fats as the primary energy source. Moderate-intensity exercise enhances lactate utilization and tolerance and improves its clearance through increased expression of MCT transporters. At this intensity, important mitochondrial and vascular adaptations continue to occur, leading to improved endurance. In turn, high-intensity exercise primarily improves VO_2__max_, maximal speed, and the ability to perform at the limits of physiological capacity by inducing central adaptations (e.g., increased cardiac output) and metabolic adaptations such as enhanced mitochondrial efficiency, greater muscle buffering capacity, and increased activity of glycolytic enzymes (Faiss et al., 2013; Matomäki, 2025). Appropriate control of training intensity and volume is key to achieving peak performance and avoiding excessive overloading while eliciting the intended adaptations (Grzebisz, 2020). However, clear guidance on how to establish training intensity zones under hypoxic conditions is currently lacking. This gap in the literature represents a significant limitation in the field.

The anaerobic threshold (AT) is one of the most important physiological markers in endurance sports, commonly used to assess athletic performance and to determine training intensity zones (Faude et al., 2009; Zhong et al., 2025). Over the past several decades, various concepts for determining the AT have been developed, primarily based on monitoring increases in La concentration or changes in respiratory variables during graded exercise tests (GXTs). Another key method for evaluating aerobic performance is the determination of the maximal lactate steady state (MLSS). The MLSS is defined as the highest exercise intensity that can be sustained over time without a progressive increase in blood La concentration. At or below this intensity, lactate production and clearance remain balanced, whereas exercising above it results in lactate accumulation due to production exceeding removal (Billat et al., 2003). By definition, the MLSS is identified when La increases by less than 1 mmol/L between the 10^th^ and the 30^th^ minute of a constant-intensity effort (Snyder et al., 1994). Determining the MLSS is a time-consuming process, as it requires several separate exercise tests, which may interfere with the planned training cycle. For this reason, the literature often compares the MLSS with other methods of assessing the AT, such as the ventilatory threshold or the lactate threshold (Płoszczyca et al., 2020).

Hypoxia decreases the exercise intensity at which the anaerobic threshold (AT) occurs, as confirmed by previous studies—including a 12–19% reduction in power output at the AT without changes in the heart rate at 3000 m a.s.l. (Faulhaber et al., 2021) and a 15% decrease in running velocity accompanied by a 5 beats·min⁻^1^ reduction in the heart rate at 2500 m a.s.l. (Friedmann et al., 2004). At the same time, blood lactate concentration remains unchanged, a finding also supported by more recent analyses (Beever et al., 2024). Although the shift of the AT under hypoxic conditions has been relatively well documented, data on the effects of moderate hypoxia (FiO_2_ = 14.5–16%) on the MLSS are still lacking. To date, only one study (Beever et al., 2024) has attempted to analyze the physiological responses associated with the MLSS under hypoxic conditions. At 2222 m a.s.l. (normobaric hypoxia), a significant reduction was observed in both power output at the MLSS (WRMLSS) by 9% and oxygen uptake at the MLSS (VO_2_MLSS) by ~12%. No significant changes were noted in the HR at the MLSS (HRMLSS), La concentration, or pulmonary minute ventilation (V̇E). However, it should be emphasized that all measurements were performed at 1111 m a.s.l. under natural conditions, while sea-level conditions were simulated using a hyperoxic breathing mixture. Moreover, participants had been previously acclimatized to the altitude, which may have influenced the results and highlights the need for verification under true normoxic conditions.

Therefore, the aim of our study was to evaluate the effects of normobaric hypoxia at FiO_2_ = 16.5% (~2000 m a.s.l.; H2000) and FiO_2_ = 14.5% (~3000 m a.s.l.; H3000) on the WR, VO_2_, the HR, selected cardiorespiratory variables, and La concentration during exercise performed at the intensity corresponding to the MLSS. Based on previous reports, we hypothesized that during exercise at MLSS intensity under normobaric hypoxia (1) the WRMLSS and VO_2_MLSS would be significantly reduced, and (2) the HRMLSS would remain unchanged.

## Methods

### Participants

Sixteen trained male cyclists (age: 30 ± 5 years; body height: 180.5 ± 8.0 cm; body mass: 75.2 ± 9.0 kg; body fat content: 10.2 ± 2.0%; VO_2max_: 57.0 ± 6.0 ml/kg/min) took part in the study. An a priori power analysis was conducted using G*Power 3.1 (Faul et al., 2007). Assuming a repeated-measures ANOVA, α = 0.05, power (1–β) = 0.80, and effect size f = 0.40, the required sample size was n = 12. Considering an anticipated dropout rate of approximately 30%, the planned recruitment target was set to n = 16. All participants were familiar with the laboratory testing procedures and had up-to-date medical examinations confirming no contraindications to exhaustive exercise in a hypoxic environment. Written informed consent was obtained from each participant prior to enrollment. All participants successfully completed every stage of the study. The inclusion criteria were as follows: at least 4 years of training experience, VO_2__max_ > 50 ml/kg/min, a minimum 6-month break from the last high-altitude training camp, and age between 25 and 40 years. The exclusion criterion was premature termination of the exercise test or failure to complete any stage of the study. The research project was conducted in accordance with the Declaration of Helsinki and approved by the Ethics Committee for Scientific Research at the Opole Medical School, Opole, Poland (approval code: KB/270/FI/2020; approval date: 17 July 2020).

### Measures

**Figure 1:**
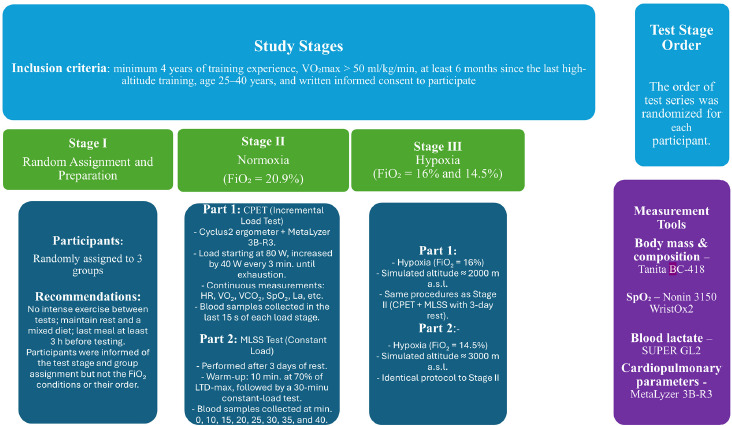
Scheme of the research protocol.

The experiment consisted of three test stages conducted under different conditions: S1 (normoxia, N), S2 (normobaric hypoxia: 2000 m, H2000), and S3 (normobaric hypoxia: 3000 m, H3000) (Figure 1). The fraction of inspired oxygen (FiO_2_) differed across the stages: S1: FiO_2_ = 20.9%, S2: FiO_2_ = 16.5%, and S3: FiO_2_ = 14.5%. The order of test stages was randomized for each participant using an Excel random number generator. All tests were performed in a normobaric hypoxic chamber (AirZone, AirSport, Poland). Participants were not informed of the actual conditions inside the chamber (normoxia or hypoxia), and they were also not informed of their heart rate or the power output during the tests. Throughout all tests, atmospheric conditions were controlled and kept constant: temperature 19–20°C, relative humidity 45–50%, carbon dioxide concentration 700–800 ppm, and oxygen fraction according to the experimental condition. In each stage, participants first performed an incremental cardiopulmonary exercise test (CPET). After a 3-day recovery period, they completed a set of constant-intensity efforts to determine the MLSS. The interval between particular stages (S1, S2, and S3) was 10 days. The methodology was consistent across all stages and all testing sessions were conducted at the same time of the day to minimize circadian variability. Participants were instructed to refrain from intense physical activity between sessions, to rest adequately, and to maintain a mixed diet. Their last meal was to be consumed no later than 3 hours before testing.

### Design and Procedures

In the first test session of each stage (S1, S2, or S3), body mass and body composition were assessed in the laboratory between 7:00 and 7:30 a.m., before breakfast. Body height was measured with an anthropometer to the nearest 0.5 cm, and body mass and body fat were estimated using bioelectrical impedance analysis (BC-418, Tanita, Japan).

At each stage, 3 h after consuming a light mixed meal, participants performed a graded exercise test to volitional exhaustion on a Cyclus2 cycle ergometer (RBM Elektronik-Automation GmbH, Germany). The protocol began at 80 W, with increments of 40 W every 3 min until exhaustion. The heart rate (HR), pulmonary ventilation (V̇E), breathing frequency (BF), oxygen uptake (VO_2_), carbon dioxide output (VCO_2_), and the respiratory exchange ratio (RER) were continuously measured at rest (3 min before the test) and throughout exercise using a fast-response gas analyzer (MetaLyzer 3B, Cortex, Germany) with the breath-by-breath method. Before each test, the device was calibrated according to the manufacturer’s recommendations for the flow and gas sensors, using ambient air (O_2_ = 20.9%) and a reference gas, followed by control measurements of room conditions to verify accuracy under the experimental settings. The MetaSoft® Studio (Cortex, Germany) software was used to analyze the results and determine the measured variables. At the end of each workload stage (last 15 s), fingertip capillary blood samples were collected to determine blood lactate (La) concentration (SUPER GL2, Dr. Müller Gerätebau GmbH, Germany). These data were used to analyze blood lactate kinetics and to determine the individual lactate threshold using the D_max_ method (LTD-max, Cheng et al., 1992). Previous studies (Czuba et al., 2009; Płoszczyca et al., 2020) demonstrated that LT determined by the D_max_ method corresponded to the MLSS.

After a 72-h rest period, participants performed a series of constant-load efforts on the Cyclus2 cycle ergometer to determine the MLSS under the conditions of each testing stage (S1: normoxia, S2: hypoxia at 2000 m, S3: hypoxia at 3000 m). Each test began with a 10-min warm-up at an individually determined intensity corresponding to 70% of the lactate threshold obtained with the D-max method (LTD-max) for the given environmental condition, performed at a cadence of 80–90 revolutions per minute. Following the warm-up, participants completed a 30-min constant-load effort at 100% LTD-max. During the constant-load tests, the HR, V̇E, BF, VO_2_, VCO_2_, and the RER were measured continuously using a fast-response gas analyzer (MetaLyzer 3B, Cortex, Germany), while hemoglobin saturation (SpO_2_) was assessed with a 3150 WristOx2 oximeter (Nonin Medical Inc., USA). SpO_2_ was measured during the blood-sampling intervals, with a one-minute finger recording at each time point—after drying the skin and stabilizing the wrist to minimize motion artifacts—corresponding to capillary blood samples taken at rest, after the warm-up, and every 5 min during the test. When La concentration during the final 20 min of exercise did not increase by more than 1 mmol/L, the test was repeated after a 3-day rest with the load increased by 20 W. Tests were continued until the final 20 min of the workload caused ΔLa to exceed 1 mmol/L; in such cases, the previous workload was considered the MLSS for that condition. The MLSS workload was typically determined after 2 (up to 3) tests performed under each condition, which was made possible by the previously conducted incremental test used to determine the workload corresponding to LTD-max, from which the MLSS test was initiated (Czuba et al., 2009, Płoszczyca et al., 2020).

### Statistical Analysis

The primary endpoint of the study was the WRMLSS. At the time of study planning, no published data were available describing the expected magnitude of change in the WRMLSS under hypoxic conditions. Therefore, the a priori power analysis was informed by previous literature reporting hypoxia-related changes in aerobic performance variables in athletes (e.g., power output, VO_2__max_) and by our pilot observations (Mollard et al., 2007; Płoszczyca et al., 2021). Based on these sources, we assumed a large effect size (Cohen’s f = 0.40) for the effect of hypoxia on the WRMLSS.

Data were collected and processed using Statistica software (StatSoft, Tulsa, OK, USA). The Shapiro-Wilk test was first used to test the normal distribution of variables and the Levene’s test was used to check the homogeneity of variance. When the assumptions for parametric testing were met, a one-way repeated-measures ANOVA was performed. Before conducting the repeated-measures ANOVA, Mauchly’s tests were performed to assess the assumption of sphericity. All variables met the sphericity assumption (*p* > 0.05), allowing the use of standard repeated-measures ANOVA without applying any corrections. Effect sizes (partial η^2^) were calculated for all significant ANOVA results, and 95% confidence intervals (CIs) for the partial η^2^ were reported. When significant differences were found by ANOVA, the Bonferroni post hoc test was used. Statistical significance was set at *p* < 0.05 for all comparisons.

## Results

Statistical analysis revealed significant differences for the WR/kgMLSS (*p* = 0.0001, F = 105.657, partial η^2^ = 0.876 (95% CI: 0.769–0.916)), the WRMLSS (*p* = 0.0001, F = 110.546, partial η^2^ = 0.881 (95% CI: 0.777–0.919)), VO_2_MLSS (*p* = 0.00001, F = 23.339, partial η^2^ = 0.609 (95% CI: 0.343–0.732)), VO_2_/kgMLSS (*p* = 0.00001, F = 24.283, partial η^2^ = 0.618 (95% CI: 0.356–0.739)), oxygen pulse at the MLSS (O_2_-pulseMLSS) (*p* = 0.00001, F = 27.489, partial η^2^ = 0.647 (95% CI: 0.396–0.759)), SpO_2_ (*p* < 0.0001, F = 191.84, partial η^2^ = 0.927 (95% CI: 0.863–0.951)), ventilatory equivalent for carbon dioxide (V̇E/CO_2_MLSS) (*p* = 0.00019, F = 11.562, partial η^2^ = 0.435 (95% CI: 0.143–0.605)), and blood lactate concentration at the 30^th^ min of the MLSS (av La 30′MLSS) (*p* = 0.00001, F = 18.680, partial η^2^ = 0.555 (95% CI: 0.273–0.693)).

**Figure 2:**
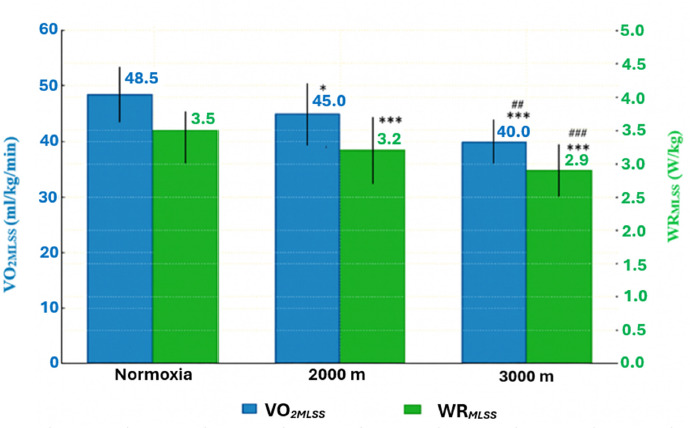
Power output (WRMLSS) and oxygen uptake (VO_2_MLSS) associated with the MLSS under hypoxic conditions. All values are presented as mean ± SD. * p < 0.05; *** p < 0.001 (significant differences between normoxia and hypoxia), ## p < 0.01; ### p < 0.001 significant differences between hypoxia conditions (H2000 vs. H3000)

Post hoc analysis showed that in H2000 and H3000, the WRMLSS and the WR/kgMLSS decreased significantly by ~9.3% and ~18.50% compared with normoxia (*p* = 0.0001). The analysis also showed a significant decrease in these variables under hypoxic conditions (H2000 vs. H3000) by 10.1% (*p* = 0.0001; Table 1, Figure 2). There was also a significant decrease in both the absolute and relative VO_2_MLSS values between N and H2000 by ~7.2% (*p* = 0.019). Between N and H3000, significant decreases were also observed for VO_2_MLSS and VO_2_/kgMLSS (~17%; *p* = 0.00001 and *p* = 0.00001, respectively). The data also indicated a significant decrease in these variables under hypoxic conditions (H2000 vs. H3000) by 10.4% (*p* = 0.001, Table 1).

**Table 1:** Power output, blood lactate concentration, and cardiorespiratory variables associated with the MLSS during exercise under normoxia and hypoxia. All values are presented as mean ± SD.

Variable	Normoxia	H2000	H3000
WR_MLSS_ (W)	261.88 ± 36.28	237.50 ± 35.59***	213.44 ± 26.31***###
WR/kg_MLSS_ (W)	3.51 ± 0.51	3.18 ± 0.52***	2.86 ± 0.36*** ###
VO_2MLSS_ (ml/min)	3622.50 ± 457.87	3361.88 ± 631.13*	3011.25 ± 479.39*** ##
VO_2_/kg_MLSS_ (ml/kg/min)	48.47 ± 5.91	44.93 ± 8.44*	40.06 ± 4.51*** ##
VO_2_/WR_MLSS_ (ml/W)	13.87 ± 0.87	14.10 ± 1.12	14.10 ± 1.32
HR_MLSS_ (bpm)	161.75 ± 11.04	162.81 ± 9.27	162.50 ± 8.91
O_2_-pulse_MLSS_ (ml/beat)	22.50 ± 3.24	20.68 ± 3.81**	18.60 ± 3.24*** ##
V̇E_MLSS_ (l/min)	102.88 ± 13.22	98.40 ± 18.38	97.86 ± 13.96
V̇E/CO_2MLSS_	29.58 ± 2.30	30.51 ± 3.55	32.59 ± 3.21*** ##
SpO_2_ (%)	94.88 ± 1.63	84.81 ± 2.56***	79.19 ± 3.49*** ###
av La_30' MLSS_ (mmol/l)	3.45 ± 1.03	4.71 ± 1.04**	4.64 ± 1.32**

Abbreviations: H2000: normobaric hypoxia (FiO_2_ = 16.5%, ~2000 m); H3000: normobaric hypoxia (FiO_2_ = 14.5%, ~3000 m); WRMLSS: power output at the MLSS; VO_2_MLSS: oxygen uptake at the MLSS; VO_2_/kgMLSS: relative oxygen uptake at the MLSS; VO_2_/WRMLSS: oxygen uptake to power output ratio at the MLSS; HRMLSS: heart rate at the MLSS; O_2_-pulseMLSS: oxygen pulse at the MLSS; V̇EMLSS: minute ventilation at the MLSS; V̇E/CO_2_MLSS: ventilatory equivalent for carbon dioxide (ratio of minute ventilation to CO_2_ output); SpO_2_: hemoglobin saturation; av La30′MLSS: blood lactate concentration at the 30^th^ min of the MLSS; * p < 0.05; ** p < 0.01; *** p <0.001: significant differences between normoxia and hypoxia; # p < 0.05; ## p < 0.01; ### p <0.001: significant differences between hypoxia conditions (H2000 vs. H3000)

These changes also led to a significant decrease in O_2_-pulseMLSS, despite the absence of significant changes in HR values. Under H2000 conditions, O_2_-pulseMLSS decreased by 8.1% (*p* = 0.005), whereas under H3000 conditions it decreased by 17.3% (*p* = 0.00001) relative to normoxia. A significant reduction (by 10.1% *p* = 0.0012) in O_2_-pulseMLSS also occurred between the hypoxic conditions (H2000 vs. H3000)

In contrast, a significant increase in VE/CO_2_ values occurred only under hypoxic conditions corresponding to 3000 m (H3000), showing a 10.2% (*p* = 0.0001) rise relative to normoxia. These values were also significantly higher compared with those at 2000 m (H2000) by 6.8% (*p* = 0.0086).

A significant increase in av La 30′MLSS was also observed between N and H2000 (+36.52%; *p* = 0.00013), as well as between N and H3000 (+34.49%; *p* = 0.00016; Table 1). Moreover SpO_2_ showed a statistically significant drop between N and H2000 (−10.61%; *p* < 0.0001), N and H3000 (−16.43%; *p* < 0.0001), and H2000 and H3000 (−6.62%; *p* < 0.0001). The greater the hypoxia, the larger the reduction in SpO_2_ (Table 1).

## Discussion

The existing literature provides limited data on the effects of normobaric hypoxia on exercise variables at the MLSS and on the associated cardiorespiratory responses. Accordingly, the present findings make an important contribution to advancing knowledge in this area. A key outcome of this study is the demonstration that significant reductions in power output, along with alterations in cardiorespiratory variables related to the MLSS in trained cyclists, occur under exposure to normobaric hypoxia equivalent to an altitude of 2000 and 3000 m a.s.l. (FiO_2_ = 16.5% and 14.5%).

### Effects of Hypoxia on the Workload (Power Output) and Blood Lactate Concentration at the MLSS

In the present study, both H2000 and H3000 significantly reduced the WRMLSS by 9.3% and 18.5%, respectively. These findings are consistent with results reported by Beever et al. (2024) who observed an approximately 9% reduction in the WRMLSS already at an altitude of 2222 m a.s.l., which is consistent with the outcomes of our research. However, it is important to highlight certain methodological differences between the studies, particularly the combination of hypobaric and normobaric hypoxia used by Beever et al. (2024). Furthermore, that study did not specify whether the unusually high peak RER values (1.27) were recorded during exercise or recovery, which complicates the assessment of their accuracy.

Among the primary causes of the reduced WRMLSS under hypoxic conditions is the decrease in SpO_2_. In hypoxia, when oxygen availability is limited, reductions in PaO_2_ and SpO_2_ occur, leading to impaired aerobic capacity and exercise performance (Mollard et al., 2007; Płoszczyca et al., 2021; Wehrlin and Hallen, 2006). The decline in aerobic capacity becomes more pronounced with greater hypoxic severity, although the extent of this decline shows considerable interindividual variability (Chapman, 2013). It has been suggested that reductions in SpO_2_ trigger a central nervous system (CNS) response which limits exercise intensity to maintain adequate arterial oxygen saturation and protects against myocardial ischemia and dysfunction of vital organs (Noakes, 2001). In the present study, SpO_2_ during MLSS testing decreased significantly to 84.8% at FiO_2_ = 16.5% (H2000) and to 79.2% at FiO_2_ = 14.5% (H3000). According to Amann and Calbet (2008), hypoxemia and hypocapnia may reduce the cerebral blood flow when SpO_2_ falls below 82%. In our study, under H2000 and H3000 conditions, the reduction in SpO_2_ was accompanied by a decrease in the WRMLSS. Our results indicate, however, that this impairment occurred already at SpO_2_ of approximately 84%, suggesting that a CNS-mediated response may be triggered at higher saturation levels than previously proposed, thereby limiting the athletes’ capacity to generate power and contributing to faster fatigue accumulation. These findings underscore the importance of monitoring SpO_2_ during hypoxic training, as even moderate desaturation may compromise power output at intensities corresponding to the MLSS

Another potential mechanism underlying the reduction of the WRMLSS under hypoxic conditions is the acceleration of anaerobic glycolysis caused by reduced O_2_ availability. This process leads to an increase in La concentration, as well as greater accumulation of H⁺ ions and inorganic phosphate compared with normoxia. Consequently, excitation-contraction coupling within muscle fibers is impaired, which has been identified as a factor contributing to loss of tension during high-intensity exercise (Amann and Calbet, 2008). It is also noteworthy that during exercise under hypoxic conditions, catecholamines (particularly adrenaline and noradrenaline) further stimulate anaerobic glycolysis, resulting in elevated La production. Thus, sympathetic nervous system activation plays a key role in the increased La and H⁺ concentrations (Piotrowicz et al., 2023).

In the present study, a significant increase in blood La concentration was observed at H2000 (+36.52% compared with N) and H3000 conditions (+34.49% compared with N), accompanied by a significant reduction in the WRMLSS. In a recent study, Park et al. (2022) reported that La concentration increased by ~36% during submaximal exercise (60% VO_2__max_) at 3000 m compared with normoxia. The authors attributed this response to reductions in SpO_2_, sympathetic nervous system activation, and increased adrenaline release, which together enhanced glycolytic activity. Similar conclusions were presented by Sumi et al. (2018) who observed a significantly greater rise in La concentration under hypoxia (FiO_2_ = 14.5%) than in normoxia during interval endurance exercise (10 × 3 min at 95% V̇O_2__max_) followed by 30 min of continuous running at 85% V̇O_2__max_.

### Effects of Hypoxia on Cardiorespiratory Variables

In the present study, hypoxia at H2000 and H3000 resulted in a significant reduction in both relative and absolute VO_2_MLSS values (7.2% and 17%) compared with normoxia. We attribute this primarily to the lower power output generated at the MLSS. These changes are mainly associated with the markedly reduced O_2_ availability during exercise (as reflected by the significant decline in SpO_2_), which is consistent with observations reported by other authors (Lawler et al., 1988), as well as with peripheral muscle fatigue. Increased acidosis, a high rate of phosphocreatine hydrolysis, and the accumulation of inorganic phosphate stimulate afferent feedback from muscle fibers, leading the nervous system to reduce the recruitment of motor units and, consequently, the number of active muscle fibers (Amann and Dempsey, 2016).

In a previous study, Beever et al. (2024) reported a significant reduction in VO_2_MLSS of approximately 12% at an altitude of 2222 m a.s.l. In our study, the percentage change in VO_2_MLSS was smaller compared with the findings of Beever et al. (2024), amounting to ~7% at 2000 m, whereas at 3000 m VO_2_MLSS decreased by 17%, which closely corresponds to the ~18.5% decline in the WRMLSS. It is worth emphasizing again that the study by Beever et al. (2024) was conducted at a natural altitude of 1111 m a.s.l. (hypobaric hypoxia), combined with a hypoxic breathing mixture, which in our view had a substantial impact on the results. The differences between physiological responses to normobaric and hypobaric hypoxia continue to be debated in the literature. Their impact on the body remains inconclusive. Initially, it was assumed that the only difference between the two conditions was the partial pressure of oxygen (PO_2_), responsible for inducing equivalent physiological changes. However, Coppel et al. (2015) demonstrated that differences exist, for example, in cardiorespiratory variables, which may influence outcomes. Similarly, Vinetti et al. (2024) found that hypobaric hypoxia was associated with higher ventilation, a lower respiratory exchange ratio, and higher VO_2__max_ compared with normobaric hypoxia—results that are consistent with those of Angeli et al. (2019).

In our study, no significant changes in V̇E were observed during the MLSS under hypoxic conditions, despite the lower mechanical work performed in hypoxia. V̇EMLSS remained at a similar level to normoxia, even though WRMLSS was significantly reduced. Bouissou et al. (1987), in a study conducted at 3000 m a.s.l. at 60% VO_2__max_, reported lower VO_2_ without changes in V̇E or the HR, suggesting an increased ventilatory cost under hypoxic conditions. In general, hypoxia induces an increase in V̇E as an adaptive response to oxygen deficiency in the tissues (Czuba et al., 2018). Similarly, as with V̇EMLSS, hypoxia did not affect the HRMLSS. Despite significant reductions in the WRMLSS, VO_2_MLSS, and SpO_2_ at H3000, the HRMLSS remained unchanged. These findings are consistent with previous studies assessing the effects of hypoxia on the cardiorespiratory system (Beever et al., 2024). For example, Burtscher et al. (2006), in two hypobaric hypoxia exercise tests lasting 5 and 50 min at 3200 m a.s.l., found no significant changes in the HR, although the WR decreased significantly by ~11% in both tests.

### Practical Recommendations and Training Implications

When setting out to achieve specific training goals, it is crucial to exercise at a precise intensity level. At the MLSS or below, La production and clearance remain balanced, while higher-intensity exercise causes La accumulation due to production exceeding removal, as indicated in the introduction. Training at MLSS intensity induces meaningful physiological adaptations, as demonstrated by Ferreira et al. (2007) who observed resting bradycardia (21%) and a 28% improvement in running performance in mice following an eight-week MLSS program. In long-distance runners, MLSS-based training leads to small increases in MLSS speed and VO_2__max_, yet importantly extends time to exhaustion by approximately 50%. This type of training enhances endurance by improving lactate clearance capacity, while lactate concentration at the MLSS itself may remain unchanged (Billat et al., 2003; Czuba et al., 2009).

Unplanned exceeding of MLSS intensity may lead to different physiological adaptations to exercise and, in prolonged effort, to profound fatigue or overtraining (Sitko et al., 2025). The present study demonstrated that under hypoxic conditions the MLSS occurs at lower exercise intensities than in normoxia and depends on changes in the altitude/level of hypoxia, which is crucial for effective training. Maintaining the pace or power (WR) at the MLSS under hypoxic conditions without referencing normoxic values will result in rapidly increasing fatigue already at an altitude of 2000 m. Moreover, understanding how the MLSS shifts with the altitude is crucial for determining an optimal warm-up, pacing, and intensity strategy during competition, especially in cycling, where precise control of exercise intensity based on power output is essential. Precisely adjusting warm-up and race intensities to MLSS shifts relative to the altitude at which the athlete is competing helps prevent premature increases in anaerobic glycolysis, reduces the risk of early fatigue, and supports achieving the best possible performance under hypoxic conditions.

Training under hypoxic conditions with the accurately determined MLSS may enhance sea-level performance. This notion is supported by our previous findings (Czuba et al., 2011, 2018) which demonstrated that cyclists’ training in hypoxia at intensities close to the lactate threshold determined using the D_max_ method—physiologically corresponding to the MLSS domain—achieved improvements in aerobic capacity and endurance performance. Therefore, although direct evidence on MLSS-specific hypoxic training remains limited, the available findings suggest that maintaining MLSS-related workloads in hypoxia may be an effective strategy for enhancing sea-level performance.

Based on our findings and available literature, we recommend adjusting training intensity during hypoxic exercise by reducing the WRMLSS relative to normoxia by ~10% at 2000 m a.s.l. and by ~20% at 3000 m a.s.l. These adjustments are consistent with the observed reduction in VO_2__max_ and allow for comparable metabolic loads to those at the sea level, thereby minimizing the risk of excessive physiological stress. Excessive exercise intensity exceeding the WRMLSS (e.g., WRMLSS + 20W) frequently resulted in test termination by participants, confirming the need for precise adjustment of training loads. Similar observations regarding reduced exercise tolerance in hypoxia have been reported by Beever et al. (2024). In situations where direct monitoring of power output is not possible, the HR is recommended as an alternative indicator of training intensity. The absence of significant changes in the HRMLSS under moderate hypoxia (2000–3000 m a.s.l.) suggests that HR zones determined under normoxic conditions can also be applied during training in hypoxic environments.

### Limitations of the Study

One of the main limitations of this study is that MLSS changes were analyzed exclusively under normobaric hypoxia. In the literature, there is an ongoing debate about potential differences in the physiological responses of the body to normobaric versus hypobaric hypoxia, which may affect the interpretation of the present findings (Coppel et al., 2015). Another limitation was the inability to provide constant accommodation for participants during the experiment, which restricted full control over their diet, recovery, and the actual training loads. Additionally, assessing intra-individual variability could provide further insight into the consistency of physiological responses across participants, thereby strengthening the interpretation of the results. Furthermore, our study included just one sports discipline and only male participants, which limits the generalizability of the results. Future research should therefore be extended to women and to other endurance disciplines, such as long-distance running, cross-country skiing, and rowing, while accounting for their specific exercise characteristics. It would also be valuable to conduct studies in female athletes and across different training levels, which would enable a more precise characterization of adaptive responses in diverse populations.

## Conclusions

The results of the present study demonstrate that exposure to normobaric hypoxia equivalent to 2000 and 3000 m a.s.l. leads to a significant reduction in VO_2_MLSS (7.2% and 17%) and the WRMLSS (9.3% and 18.5%). At the same time, HR values remained unchanged regardless of the level of hypoxia during exercise performed at MLSS intensity. Therefore, in situations where individual WRMLSS values are not available, it is reasonable to prescribe training intensity in moderate hypoxia based on HR zones determined under normoxic conditions. Moreover, for precise adjustment of training loads at an altitude of 2000 and 3000 m a.s.l., it is recommended to reduce threshold power values by 10% and 20%, respectively, relative to those established under normoxia. Future research should focus on different types of hypoxia, more rigorously controlled living conditions, assessments of intra-individual variability, and more diverse athlete populations (including women and athletes from various endurance disciplines) to enable a more precise characterization of physiological adaptations.
